# Forecasting ward-level bed requirements to aid pandemic resource planning: Lessons learned and future directions

**DOI:** 10.1007/s10729-023-09639-2

**Published:** 2023-05-18

**Authors:** Michael R. Johnson, Hiten Naik, Wei Siang Chan, Jesse Greiner, Matt Michaleski, Dong Liu, Bruno Silvestre, Ian P. McCarthy

**Affiliations:** 1 Beedie School of Business, Simon Fraser University, Vancouver, Canada; 2 Department of Medicine, University of British Columbia, Vancouver, Canada; 3 Department of Medicine, Providence Health Care, Vancouver, Canada; 4 Department of Medicine, Vancouver General Hospital, Vancouver, Canada; 5 Land and Food Systems, University of British Columbia, Vancouver, Canada; 6 Asper School of Business, University of Manitoba, Winnipeg, Canada; 7 Luiss Guido Carli, Rome, Italy

**Keywords:** Forecasting, Machine learning, COVID-19, Ward-level forecasting, Traffic Control Bundling, Pandemic resource planning

## Abstract

During the COVID-19 pandemic, there has been considerable research on how regional and country-level forecasting can be used to anticipate required hospital resources. We add to and build on this work by focusing on ward-level forecasting and planning tools for hospital staff during the pandemic. We present an assessment, validation, and deployment of a working prototype forecasting tool used within a modified Traffic Control Bundling (TCB) protocol for resource planning during the pandemic. We compare statistical and machine learning forecasting methods and their accuracy at one of the largest hospitals (Vancouver General Hospital) in Canada against a medium-sized hospital (St. Paul’s Hospital) in Vancouver, Canada through the first three waves of the COVID-19 pandemic in the province of British Columbia. Our results confirm that traditional statistical and machine learning (ML) forecasting methods can provide valuable ward-level forecasting to aid in decision-making for pandemic resource planning. Using point forecasts with upper 95% prediction intervals, such forecasting methods would have provided better accuracy in anticipating required beds on COVID-19 hospital units than ward-level capacity decisions made by hospital staff. We have integrated our methodology into a publicly available online tool that operationalizes ward-level forecasting to aid with capacity planning decisions. Importantly, hospital staff can use this tool to translate forecasts into better patient care, less burnout, and improved planning for all hospital resources during pandemics.

## Highlights

- Ward-level forecasting methods can aid hospital staff in expanding and contracting the size of COVID-19 wards for improved resource management.
- Statistical and machine learning forecasting methods can provide better accuracy in anticipating required beds in both confirmed COVID-19 positive and patient-under-investigation (PUI) wards than human decision-making alone.
- The upper 95% prediction interval of an ARIMAX forecasting method improves forecasting accuracy by approximately two-fold compared to planned capacity levels set by hospital staff.
- Using time-lagged epidemiology information (community positivity rates and daily reported COVID-19 cases) can improve forecast accuracy during inflection periods (increasing or decreasing bed demand) in hospital wards, particularly for larger hospitals that emulate community-related health impacts.
- A forecasting planning tool has been built into a publicly available online tool that operationalizes forecasting and aids in planning decisions at the ward level to support pandemic relief. The tool can be accessed here: https://stpaulhospital.shinyapps.io/hospital_planning/

## Introduction

The COVID-19 pandemic has put a significant strain on healthcare resources worldwide, often challenging the capacity of hospitals and placing excessive stress and anxiety on healthcare workers – the greatest asset of our healthcare systems. This is also the case in Canada, where six to seven pandemic waves have strained hospital resources. According to recent polls, 70% of surveyed Canadian healthcare workers reported mental fatigue, including experiencing sleep deprivation and burnout [1, 2]. The need to cohort hospitalized patients based on their COVID-19 status has further increased the complexity of healthcare system operations and added to healthcare worker stress.

During the pandemic, many forecasting methods have been applied to understand the growth and decline of the total number of people infected with COVID-19 across different jurisdictions. However, there is a dearth of research investigating forecasting methods at a more granular level, including specific hospitals [3, 4] and even specific departments, wards, and units [5]. In the context of a pandemic, forecasting strategies for specific wards or hospital units could assist decision-makers with short-term planning in ways that city or region-wide forecasts cannot. Hospitals in different areas with the same jurisdiction could experience differing demand patterns for hospital beds for several reasons, including sociodemographic variation, localized outbreaks, attitudes toward following public health measures, and the concentration of similar facilities [6, 7]. From a supply perspective, hospital and ward-specific factors could result in day-to-day changes in bed availability, such as staffing shortages and limitations in physical space.

In the world of time-series forecasting, the two popular categories of forecasting methods used today are traditional “statistical” time-series methods and more recently applied “algorithmic” machine learning (ML) forecasting methods [8]. This paper investigates if statistical or ML forecasting methods can provide improved accuracy in anticipating the number of beds required in COVID-19 wards while functioning within a modified Traffic Control Bundling (TCB) protocol for resource planning during the pandemic. TCB protocols have been implemented in hospitals worldwide during the earliest stages of the COVID-19 pandemic. Operationally, their goal is to minimize nosocomial transmissions by separating known positives, unknowns (PUI), and known negatives to minimize nosocomial outbreaks and provide capacity planning and resource management of COVID-19 wards [9].

The main contribution of this paper is our proposal for a forecasting system that enables an accurate short-term prediction (up to approximately 7 days) of bed requirements within hospital wards during the early stages of pandemic resource planning. This forecasting system is applicable in situations where pandemic cases are not monotonically increasing or decreasing, but rather COVID-19 cases are expected to experience moderate expansion and contraction over time and can be cared for within the existing capacity of local healthcare facilities.^1^ The forecasting system has effectively been applied to the earliest stages of pandemic resource planning when the greatest element of diagnostic uncertainty (e.g., vaccinations were unavailable, and patients were separated within hospital wards into 3 distinct patient cohorts^2^). The forecasting system can be adapted for future outbreaks of a pandemic or other viral outbreaks where ward-level hospital care and staffing are strained in terms of patient loading. To achieve this main contribution our research includes:

- An evaluation of the accuracy of statistical and machine learning forecasting methods for predicting bed requirements within COVID-19 wards during the first three waves of the COVID-19 pandemic (end of March 2020 to June 30, 2021). Statistical [10–13] and ML [14–16] forecasting have proven effective for population growth models during the COVID-19 pandemic, but no known research has explored their application to ward-level demand in hospitals. Likewise, no known literature identifies under what conditions such forecasting methods are useful and applicable to pandemic forecasting at the ward level and to what extent. We explore the benefits and the limitations of such forecasting methods for ward-level predictions using comprehensive time-series evaluation techniques comparing such methods to other forecasting methods employed in the literature.
- An evaluation of external covariates in such forecasting models including time-lagged variables that demonstrate significant correlations with bed requirements in hospital wards.
- An evaluation of the use of prediction intervals in such forecasting methods to model the uncertainty of point forecasts and their ability to anticipate swings in patient demand at the ward level of hospitals.
- The selection of the most appropriate standardized forecasting methods (statistical and ML methods) to include in an online forecasting system for ward-level predictions, that provides both point forecasts and prediction intervals to support planning decisions.
- In practical terms, this paper shows how statistical and ML forecasting methods can contribute to supporting the decision-making of ward-level bed requirements for the health of patients and the important planning of hospital employees in two hospitals in Vancouver Canada.

**Fig. 1:**
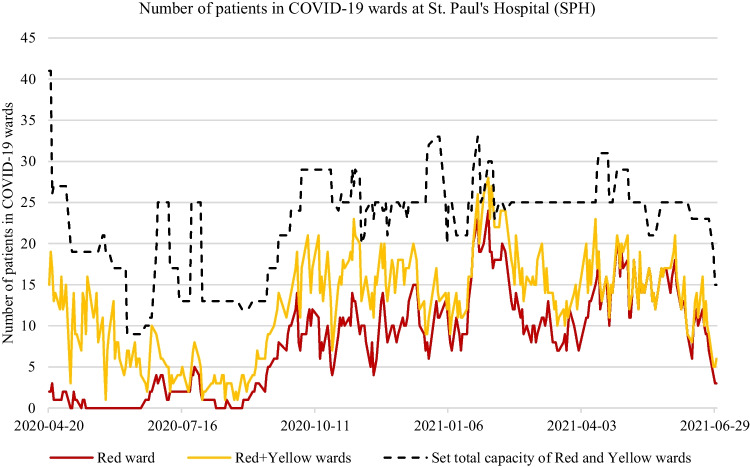
Number of COVID-19 patients at St. Paul’s Hospital from April 2020 until June 2021. The red line represents the total number of COVID-19 positive patients assigned to the Red ward. The yellow line represents the total number of patients assigned to both the Red and Yellow wards. The black dotted line represents the set capacity level of the beds allocated to both the Red and Yellow ward

**Fig. 2:**
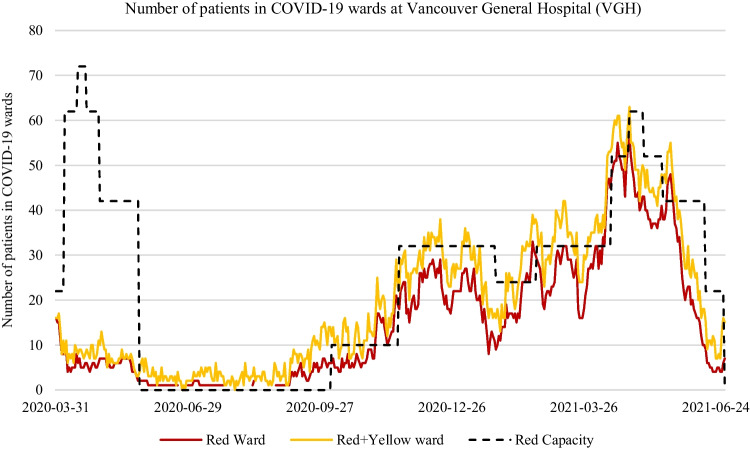
Number of COVID-19 patients at Vancouver General Hospital from March 2020 until June 2021. The red line represents the total number of COVID-19 positive patients assigned to the Red ward. The yellow line represents the total number of patients assigned to both the Red and Yellow wards. The black dotted line represents the set capacity level of the beds in the Red ward only

To make our research contribution truly beneficial, we have developed a working prototype forecasting tool that allows for “real-time” forecasting capabilities at the ward-level during a pandemic. Its purpose is to be used and operated by healthcare professionals working within hospital wards—not data scientists—and has been programmed into an online tool that is publicly available for use. Our tool forecasts bed requirements within individual COVID-19 wards and provides a planning module that predicts the likelihood that future patient demand within a given COVID-19 ward will exceed hospital set capacities within a defined time horizon. In developing this tool, we investigated univariate and multivariate methods to find the best predictors among these methods while balancing accuracy and simplicity of implementation. For simplicity we focus on three important conditions: (1) hospital staff does not require prior knowledge of forecasting methods to use it, (2) training of the forecasting method itself is not a requirement to conduct forecasting, and (3) the method should have as few input variables as possible, and these variables should be easily obtainable by hospital staff. The tool empowers physicians, nurses, and administrators to make “real-time” informed decisions when planning hospital resources, including the compounding impacts of schedules relating to urgent and elective surgical procedures.

Our paper is organized as follows. Section 2 discusses the need for research for exploring the accuracy of ward-level forecasting methods that are possibly less complicated and more accurate than current ward-level forecasting methods published in the literature. Section 3 describes the setting at the two hospitals in Vancouver, Canada during the pandemic. It also presents the data used in this study to compare alternative forecasting methods and identifies the opportunity for improvement in ward-level resource planning. Section 4 provides an overview of this study’s statistical and ML forecasting methods and the cross-validation techniques used to compare forecast accuracy. Section 5 presents the results and reflects upon the accuracy of the various forecasting methods and uncertainty levels. In Section 6, we discuss the implication of our findings and the value of our results for scholars, practitioners, and most importantly hospital staff working in COVID-19 wards. We further discuss the development of the online prototype forecasting tool to support ward-level decisions. Lastly, we provide concluding remarks and our aims for further research.

## Literature

Forecasting methods have proven effective for hospital resource management in a variety of settings, including healthcare emergency departments [17–20]; outpatient visits [21, 22]; hospital bed capacity [23, 24]; hospital services [25–27], and most recently a surge of research has focused on pandemics [3–5, 11–16, 28–48]. Given the unique circumstances presented within each country during the COVID-19 pandemic, forecast method selection and its application has been varied. The selection of forecasting methods includes statistical time-series methods [11–13, 28–30, 37, 38, 43, 48], machine learning methods [14–16, 19, 20, 27, 33, 35, 36, 39, 42, 47], simulation and queueing based methods [3, 5, 40] and combined approaches [4, 15, 33–36, 39, 41, 46]. Table 1 summarizes the similarities and differences between forecasting methods and their applications during the COVID-19 pandemic. A large majority of forecasting methods are applied within a specific geographical context at the country [11–13, 16, 28, 30, 34, 35, 37–39, 43, 44, 48], regional or local health authority level [14, 16, 29, 32, 33, 40–42, 45, 47] to predict the total demand of hospital resources in aggregate to plan for larger geographical resources such as the total number of intensive care unit (ICU) beds and ventilator demands.

**Table 1:** Forecasting Methods developed during COVID-19

Authors	Target Level Predictions	Forecasting Method(s)	Investigated Area
Alabdulrazzaq et al. (2021)	Country Level	Autoregressive integrated moving average (ARIMA)	Kuwait
Alzahrani et al. (2020)	Country Level	ARIMA	Saudi Arabia
Aslam (2020)	Country Level	Kalman Filter (KF), ARIMA, & Holt-Winters (HW)	Pakistan
Baas et al. (2021)	Hospital ICU; Hospital COVID-19 wards	Richards’ curve, Kaplan–Meier estimation, Poisson Arrival Location Model	Netherlands
Barret et al. (2020)	Provincial Level	Scenario-Based Individual Level Simulation	Ontario, Canada
Bekker et al. (2021)	Country Level	Linear Programming, Kaplan–Meier estimation, and Queuing Model	Netherlands
Bhandari et al. (2020)	Local Health Jurisdiction	Survival Analysis, Cox proportional hazard regression analysis	Rajasthan, India
Bhandary et al. (2020)	Country Level	ARIMA, exponential smoothing (ETS), & Susceptible-infectious-recovered (SIR)	Nepal
Braga et al. (2021)	State Level	Artificial Neural Networks (ANN)	Pará, Brazil
Calabuig et al. (2021)	Hospital Level	Kaplan–Meier Survival Curve	Granada, Spain
Capistran et al. (2021)	City Level (ICUs)	SEIRD Model; Bayesian	Mexico
Ceylan (2020)	Country Level	ARIMA	France, Italy, & Spain
Chakraborty and Ghosh (2020)	Country Level	ARIMA, wavelet-based Forecasting (WBF)	Canada, France, India, & South Korea
Darapaneni et al. (2021)	State Level	SIR/SEIR, Time-series Analysis (ARIMA)	Telangana, India
Garcia-Vicuña et al. (2021)	Country Level	Population Growth Models; DES	Spain
Goic et al. (2021)	Local Health Jurisdictions	Trimmed Mean to combine ARIMA/ARIMAX, ANN, and compartmental models	Chile
Hasan (2020)	Global Cumulative Cases	ANN – ensemble empirical model decomposition (ANN-EEMD) MA, & Linear Regression	World (aggregate)
Joseph et al. (2020)	Country Level	ETS & integer-valued generalized autoregressive conditional heteroskedastic (INGARCH)	Nine Countries
Kamar et al. (2021)	Country Level	Modified CHIME Model (SIR Model) ESTF Calculator, AUBMC Surge Needs Calculator	Lebanon
Khan & Gupta (2020)	Country Level	ARIMA & Nonlinear autoregressive neural network (NNAR)	India
Kufel (2020)	Country Level	ARIMA	32 European Countries
Macca et al. (2020)	Country Level	Population Growth Models	Italy
Melin et al. (2020)	Country and State Level	Multiple ensemble artificial neural network	Mexico
Perone (2020)	Country and Regional Level	ARIMA	Italy, Russia, & USA
Ribeiro et al. (2020)	State Level	ARIMA, cubist regression (CUBIST), random forest (RF), ridge regression (RIDGE), support vector regression (SVR), & stacking-ensemble learning (SEL)	Brazil
Petropoulos and Makridakis (2020)	Country Level	ETS	World (aggregate)
Singh S. et al. (2020)	Country Level	ARIMA-WBF	France, Italy, Spain, UK, & USA
Swaraj et al. (2020)	Country Level	ARIMA, NNAR, & ARIMA-NNAR	India
Toharudin et al. (2021)	Provincial Level	NNAR, Multi-layer Perceptron (MLP), Extreme Learning Machine (ELM)	Jakarta, West Java Indonesia
Wieczorek et al. (2020)	Country and Regional Levels	ANN	Several countries & regions
Yang et al. (2021)	Hospital Acute Care and ICU wards	SIR, Simulation	California, USA
Yonar H. et al. (2020)	Country Level	ARIMA & Brown/Holt linear exponential smoothing method (B/W LES)	G8 countries
Zhao et al. (2020)	Provincial and City Level	ARIMA, ARIMAX, ETS, & Susceptible-infected but undetected-infected quarantined-suspected-discharged (SEIQDR) ARIMA	China

As shown in Table 1, very few papers have investigated forecasting methods specifically to predict ward-level demand requirements during the pandemic. Yang and colleagues [3] have developed a probabilistic model that translates regional COVID-19 estimates into hospital-specific forecasts with prediction intervals. Their forecasting method relies upon the accuracy of regional forecasts to create accurate predictions at the hospital level. A common forecasting method deployed during the pandemic, and utilized by Yang and colleagues [3], are epidemiologic forecast methods such as SIR, SIR, SEIR SEIRD models that are based on a set of differential equations with defined initial conditions and adaptive parameters [49]. Such models are primarily designed to forecast large populations rather than individual hospitals [3] and are sensitive to the definitions of initial conditions and parameters [50]. Despite such challenges, Yang and colleagues [25], demonstrate accurate predictions at the hospital level using three varied assumptions about the accuracy of regional forecasts (perfect forecasts, unbiased forecasts, and biased forecasts).

Another valuable research contribution to forecasting at the hospital level comes from Garcia-Vicuna and colleagues [51]. These researchers simulate patient arrivals based on Population Growth (PG) models and subsequently use a discrete-event-simulation (DES) model to generate uncertainty of point forecasts. Likewise, Manca and colleagues [49] use a similar approach to forecasting ICU bed demand using PG models. PG models are applicable to forecasting monotonically increasing bed numbers, which never decrease over time, up to a maximum plateau level [49]. Thus, PG models are appropriate for larger geographical regions or hospitals that experience sustained growth in total hospitalizations to a maximum value; followed by a plateau, and finally, a pattern of decreasing trend to zero when the pandemic expires. Conversely, as in our study, individual wards are more likely to experience non-monotonically increasing trends where the number of patients requiring beds increases and decreases with relatively fewer fluctuations. PG models cannot be used in such scenarios [49].

Our work complements prior research on ward-level forecasting in several ways. Based on our extensive literature review and our best knowledge, no research has yet investigated the use of statistical and machine learning forecasting models to predict bed requirements at the ward-level. Although there exists plenty of research on how such models can accurately predict regional or country-level growth patterns of the pandemic, no literature has quantified the effectiveness of such classical approaches to predict ward-level demand during the earliest stages of the pandemic. Compared to classical statistical and ML forecasting methods, most ward-level forecasting methods published in the literature to date are relatively complicated. Functionally and practically speaking, such classical methods may prove to be easier to implement (for exploration and tool development) and provide as good or better accuracy for ward-level planning and decision-making. Such forecasting methods have the added benefit of using readily available data at the ward-level, which does not need to be modified from regional forecasts as previous studies have done. Given that ward-level demand will vary across different hospitals, forecasting at the point of real-time demand (without modifications to the time series) will likely provide the greatest overall accuracy of bed demand that is realized. This is analogous to forecasting retail demand for multiple consumer products across different geographical retail locations. To accurately predict the consumer demand for specific products at a single location, one needs to estimate the drivers of demand fluctuations that will depend on a single location and single SKU [52]. Further, some statistical and ML forecasting methods allow for the inclusion of covariates or external factors that can pick up on patterns outside the time series. Thus, there exists an opportunity to investigate the use of epidemiological and community-related factors, that may serve as useful inputs to such forecasting methods to help capture trend effects for improved accuracy. Lastly, no literature seems apparent that investigates the accuracy of prediction intervals of such methods as most research on ward-level forecasting methods has focused on. In this paper, we strive to fill this literature gap for researchers and practitioners alike.

## Setting

This section describes the operational contexts of the two hospitals (St. Paul’s Hospital and Vancouver General Hospital) during the first three waves of the COVID-19 pandemic in BC (approximately, from the end of March 2020 to June 2021 [53]). St. Paul’s Hospital (SPH) is considered a moderate-sized hospital in Canada with a total of 446 beds. Vancouver General Hospital (VGH) is the tenth largest hospital in Canada with a total of 926 beds [54]. Both hospitals are in Vancouver, BC residing within the Vancouver Coastal Health Authority (VCHA) which serves approximately 1.25 million of British Columbia's population of five million. Like many hospitals worldwide [9, 55–58], both SPH and VGH implemented a modified TCB protocol to maximize the use of existing healthcare resources and to mitigate the spread of COVID-19 within their hospitals during the early stages of the pandemic. As discussed in [9], a modified TCB protocol called “Red-Yellow-Green” is a system for stratifying patients into 1 of 3 risk profiles and corresponding ward spaces, named Red, Yellow, and Green probability of disease. Red spaces are for patients confirmed to have COVID-19. Green spaces are for patients with a low enough diagnostic probability for COVID-19 infection that can be cohorted as usual with universal precautions. Yellow spaces are reserved for patients with great enough diagnostic probability for COVID-19 infection that additional infection control precautions are warranted. Red and Yellow wards have the strictest infection control measures involving reduced density of patient cohorts, access restrictions for health care providers, etc. These wards represent possible locations of nosocomial spread and often require additional resources (bed spaces, altered nursing ratio/ physician ratios, increased consumption of PPE, etc.) to adequately prevent these infections. Patients admitted to the Red and Yellow wards are cared for by designated COVID teams to limit the potential spread to healthcare workers (HCWs) and patients in Green zones.

There are two important types of resource planning decisions within the modified TCB protocols at SPH and VGH that are impacted by this study. One is related to the total ward space within the hospital and the other is related to the separation of individual wards and the assigned bed and staffing numbers to each ward. The total hospital ward space of Internal Medicine at both hospitals is fixed due to hospital infrastructure that cannot be easily adjusted. One of the main applications of this research is being able to predict the possibility that the total ward space will overflow into other hospital units or beyond the capacity of the hospital. This impacts the total required number of HCWs and the functionality of hospital units beyond the COVID-19 wards. The second important ward-level decision is related to the operational expansion and contraction of individual COVID-19 wards within the total ward space. This is because bed numbers, nurses, and allied health teams are often assigned on a per ward basis to provide the necessary level of biocontainment and safety for both patients and healthcare workers, thus minimizing the potential for nosocomial transmissions. Operationally, these decisions influence the location of moveable walls and labels on the individual patient rooms to create clear demarcations between the Red, Yellow, and Green wards. Thus, ward sizes at both hospitals were interdependent: increasing or decreasing the number of beds in one ward directly impacted the size of the other wards and their required healthcare resources. At SPH and VGH, hospital decision-makers made these decisions based on their assessment of the demand (COVID-19 cases) and supply (staffing and physical space) at a particular point in the pandemic.

Figure 1 and 2 show the number of patients assigned to the Red, and the combined Red-Yellow COVID-19 wards at SPH and VGH from approximately April 2020 to June 2021.^3^ Figure 1 shows the set capacity levels (black dotted line) of the total COVID-19 beds in the combined Red and Yellow wards at SPH. Figure 2 shows the set capacity levels (black dotted line) of only the Red ward at VGH.^4^ These lines reflect the decisions of hospital staff related to COVID-19 ward expansion or contraction. A few interesting observations can be deduced from these two figures. At SPH, it is quite possible that the set capacities were too high during the pandemic, particularly in the Yellow ward. This indicates the times when possibly too many beds and respective staffing resources were assigned to individual wards. In total, the COVID-19 ward’s (Red and Yellow) total set capacity at SPH experienced on average 186% higher than the required number of beds needed. Figure 2 shows the capacity of the Red ward at VGH was set unusually high at the start of the pandemic given the uncertainty of the pandemic on hospital resources. Likewise, Fig. 2 shows the total ward-level demand at VGH was significantly higher than at SPH, having several periods of doubling or tripling of ward-level demand within a week or two. As shown in this paper, statistical and ML forecasting methods with defined uncertainty levels (80%-99%) can aid in decision support for the expansion or contraction of COVID-19 wards at these two hospitals. One way we demonstrate this in this paper is by comparing the set capacity levels of the COVID-19 wards made by hospital staff to the prediction intervals of various forecasting methods. We also apply a similar ward-level evaluation methods of forecasting methods used in literature to evaluate the accuracy of ward-level forecasting methods used in this study.

## Methods

This section discusses the data used in this study, the selection of appropriate statistical and ML forecasting methods, and the techniques used to compare their accuracy. Our research followed a traditional time-series analysis by first conducting exploratory data analyses to identify the underlying time-series characteristics of the data. Next, based on those underlying characteristics, we selected appropriate models to apply to training data sets for model estimation, identification, and validation. Lastly, we applied those forecasting methods to a testing data set, which allowed us to conduct in-sample validation to identify the accuracy of the methods using several error metrics and benchmarks.

We explored several approaches of time-series cross-validation techniques to quantify error metrics (i.e., accuracy), including using different holdout periods over the times-series and forecasting using an expanding window technique that predicts from 1 to 14 days ahead. This approach provides the best validity of how a time series model will perform to real data [59]. We used a cross-validation approach similar to that of Baas et al. [5] that validated a forecasting method during COVID-19 to predict bed occupancy in ICUs and hospital wards. This allowed for a rigorous approach to quantity forecast accuracy between various statistical and ML forecasting methods and compared accuracy to academic literature (as found by Baas et al. [5] and Yang et al. [3]) for forecasting at the hospital ward level. We also utilize a forecasting evaluation technique applied by Yang et al. [3], forecasting weekly across the periods of the pandemic with the greatest stress and volatility to measure the coverage rate (CR) and accuracy metrics of competing forecast methods.

### Data

The data used in this study are ward-level bed occupancies at SPH and VGH as discussed in Section 3. We conducted extensive exploratory data analysis to select possible forecasting methods that could prove valuable in this study. Three time series at both hospitals (occupancy in the Red, Yellow, and combined Red & Yellow wards) represent the important demand profiles that are target variables for forecasting. The three datasets (Red, Yellow, and Red and Yellow combined) at both hospitals were investigated for trend, level, and seasonal effects using various statistical tools, including time-series plots, ACF/PACFs, decomposition plots, and statistical tests for trends and seasonality. Stationary tests using augmented Dickey-Fuller (ADF), Philips Perron (PP), and Kwiatkowski–Phillips–Schmidt–Shin (KPSS) unit root tests provide overall confirmatory evidence that all three time-series from St. Paul’s Hospital are nonstationary, and stationarity is achieved using first differences. Our results found that the 3 time series at both hospitals showed little evidence of seasonality, with primary components being stationary, trend, and lagged effects – indicating all 3 time series could be modeled effectively using autoregressive techniques.

We also investigated the validity of using epidemiological and external data sources in our study. Two of the forecasting methods explored (ARIMAX and NARX) are designed to allow for external covariates to be incorporated to improve their predictive performance. Publicly available data was limited to the following epidemiological variables: the number of reported daily new cases (at both provincial and local health regions), the total number of active cases (provincial only), and daily positivity test rates (local health regions only). The data used at the local health region is Vancouver Coast Health Authority (VCHA) for both SPH and VGH. We also investigated weather data in our analysis: daily average temperature and daily total precipitation. The epidemiological data came directly from the BC Center for Disease Control [53], and the weather data was extracted from weather resources provided by the Government of Canada [60].

Tables 2 and 3 provides a heatmap comparing the bivariate correlations (Pearson’s coefficient of correlation *r*) between the internal data of the number of ward-level beds occupied at both hospitals and external variables. We conducted an extensive correlation analysis of time-lagged external variables and their relationship with hospital ward-level demand to identify optimal time-lagged effects (examples provided in Fig. 10 in Appendix 1). SPH shows a relatively lower correlation with external covariates for the Yellow and combined Red and Yellow wards, with its highest correlation between the occupancy of the Red ward and VCHA positivity (with an optimal lag of 14 days).^5^ The larger hospital (VGH) had a significantly higher correlation with ward-level demand and the external epidemiological data (using optimal lags).

**Table 2:**
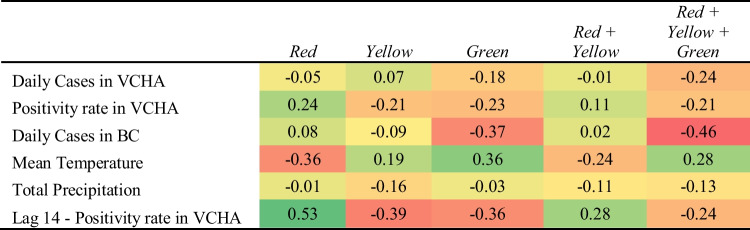
Correlations (Pearson’s coefficient of correlation *r*) between epidemiological, external data and ward occupancy at SPH from Sept. 14, 2020, to June 29, 2021

**Table 3:**
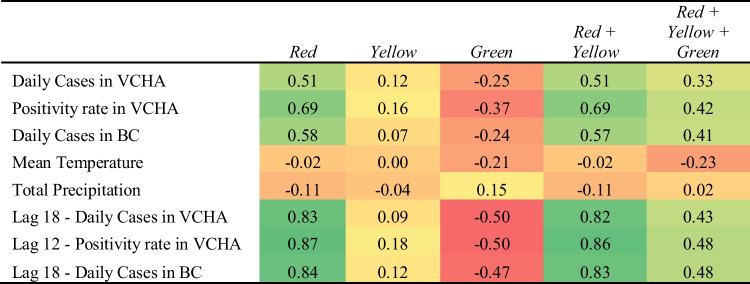
Correlations (Pearson’s coefficient of correlation *r*) between epidemiological, external data and ward occupancy at VGH from Sept. 14, 2020, to June 29, 2021

As shown in Table 3, both the Red ward and the combined Red and Yellow wards show positive bivariate correlations ranging between 0.82 and 0.86 with lagged external variables. Similarly, the number of patients in the Yellow ward at both hospitals demonstrates a relatively weaker correlation with external variables. Noticeably, correlations between community effects and the number of patients in the wards are weaker at SPH compared to VGH. We believe this is due to the ward-level demand at the larger hospital having greater ability, due to its size, to reflect a stronger association with community-level factors relative to the smaller hospital. This is also true of the lag analysis demonstrating significantly higher correlations with lagged external variables at VGH than SPH.

Table 4 shows the bivariate correlations between our 5 external variables. The daily new reported cases for BC, daily new reported cases for the VCHA, and total active cases for the BC province are all highly correlated. Given these findings, we selected to use the positivity rate in VCHA (lagged 14 days) for SPH and the daily reported new cases in the VCHA region (lagged 18 days) for VGH as an external epidemiological variable in our analysis.

**Table 4:**
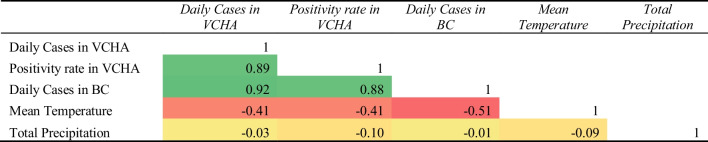
Correlations (Pearson’s coefficient of correlation *r*) between epidemiological and external from Sept. 14, 2020, to June 29, 2021

**Fig. 3:**
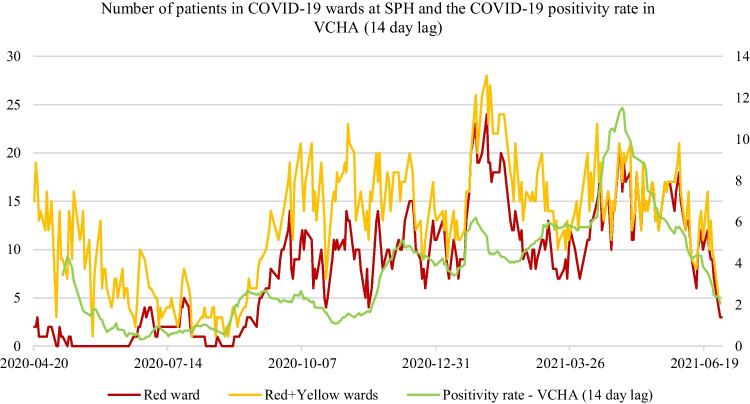
Positive correlation (*r* =  + 0.72 for Red and *r* =  + 0.54 for Red + Yellow) between the total number of patients in the COVID-19 wards at Vancouver General Hospital (VGH) (on the left vertical axis) and a 14-day lag of the COVID-19 positivity rate within the Vancouver Coastal Health Authority (VCHA) (on the right vertical axis)

**Fig. 4:**
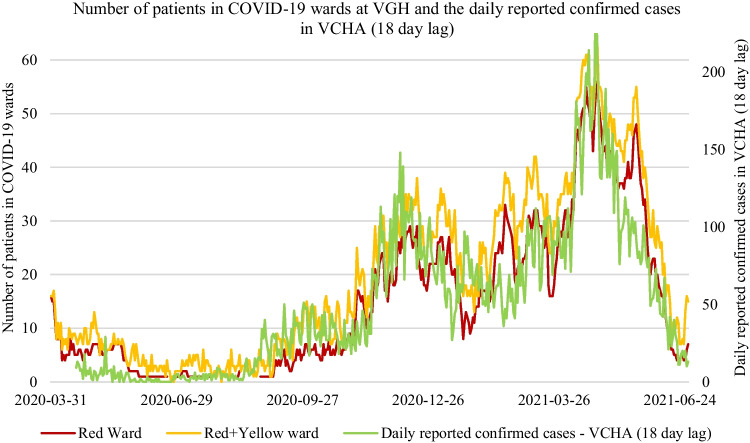
Positive correlation (*r* =  + 0.89 for Red and *r* =  + 0.90 for Red + Yellow) between the total number of patients in the COVID-19 wards at Vancouver General Hospital (VGH) (on the left vertical axis) and an 18-day lag of the daily reported confirmed cases within the Vancouver Coastal Health Authority (VCHA) (on the right vertical axis)

**Fig. 5:**
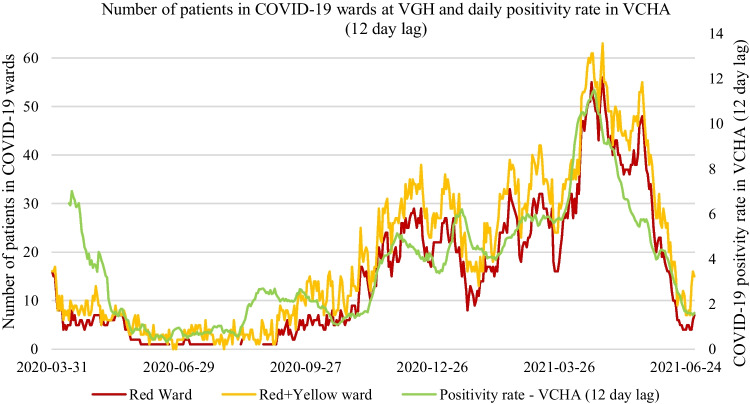
Positive correlation (*r* =  + 0.88 and *r* =  + 0.87) between the total number of patients in the COVID-19 wards at Vancouver General Hospital (VGH) (on the left vertical axis) and a 12-day lag of the positivity rate within the Vancouver Coastal Health Authority (VCHA) (on the right vertical axis)

Figure 3 shows that the number of patients in the COVID-19 wards at the smaller hospital (SPH) have a moderate correlation with daily positivity rate (optimal lag of 14 days) in the VCHA. Conversely, Figs. 4 and 5, shows the optimal lag of daily reported new cases and positivity rates in VCHA, are highly correlated with daily ward-level demand at VGH. In this paper, we investigate how such differences impact the accuracy of ward-level forecasts across a number of statistical and ML forecasting methods.

### Statistical and machine learning forecasting methods

In this subsection, we briefly provide an overview of the selection of statistical and ML forecasting methods employed in this study and provide appropriate reasoning for their use. The statistical and ML forecasting methods applied in this study are benchmarks and standards within academia and industry. For example, in the recent M4 competition [61] that publicly invites researchers and practitioners worldwide to compete in a forecast competition, all of the following statistical and ML forecasting methods used in this study were classified as standards to which competing methods were compared. Given this knowledge and the statistical properties of the time series discussed in Section 4.1, four exponential smoothing methods were investigated in this study: (1) Simple Exponential Smoothing (SES), (2) SES with a special form of drift known as the Theta method, (3) Holt’s Linear Trend Method, and (4) Holt’s Method with Dampened Trend. These methods are considered univariate models that only utilize the time-series data in the model development. ARIMA and ARIMAX were found to be highly appropriate statistical forecasting methods for ward-level forecasting in this study.^6^ ARIMA stands for autoregressive (AR) integrated (I) moving average (MA) and is based on the underlying principle that future values can be effectively predicted from a linear combination of past observations and white noise innovations (error terms). ARIMAX is a more general form of the ARIMA method that allows for the inclusion of exogenous covariates (i.e., “X”), providing the ability for multivariate time-series estimation. This technique allowed for the inclusion of epidemiological data to be modeled with the observed time series of bed occupancy within the COVID-19 wards. All statistical forecasting methods above were implemented using the “forecast” package in R developed by Rob Hyndman [62]. Interestingly, in a recent study by Makradkis and colleagues [63], these authors found that these statistical forecasting methods dominated popular ML forecasting methods in forecast accuracy across all cross-validation time horizons and all forecast error metrics. Nonetheless, we implemented one of the more accurate ML forecasting methods for time-series analysis found in comparative studies [63, 64]: a Multilayer Perceptron (MLP) neural network model that is constructed using one hidden layer. In this form, the MLP can be classified as a nonlinear autoregressive neural net (NAR). A neural network can be applied to univariate and multivariate time series using a special class called time-delayed neural networks. Likewise, a NAR with covariates of relevant epidemiological data is classified as NARX. We implemented the NAR and NARX using two different R packages (“forecast” [62] and “nnfor” [65]) to experiment with performance and accuracy. We conducted comprehensive diagnostics and validation methods of the specified models and experimented with both manual and automatic model parameter specifications. Prediction intervals for all forecasting methods were based on the cross-validation residuals derived (bootstrapping and simulation) using an expanding window procedure that is discussed in the next section.

Error metrics were calculated using standard error forecasting metrics for accuracy: (1) the mean absolute error (MAE), and (2) the root mean squared error (RMSE). The metrics were used to compare the accuracy of the forecasting methods against one another and the accuracy of the methods against the current set capacity levels of the two COVID-19 wards (Red and Yellow) at St. Paul’s Hospital. We also further validated all forecasting methods using three benchmark indicators that will be discussed.

### Evaluation methods

Two comprehensive evaluation methods are presented to validate the accuracy of our selected forecasting methods and various statistical and ML forecasting methods in general when applied to ward-level forecasting. The first is a cross-validation technique similar to Baas et al. [5], which provides an expanding window procedure developed by Hyndman and Athanasopoulos [59]. This method uses a comprehensive evaluation of the accuracy of the various statistical and ML forecasting methods against forecasting benchmarks and set capacity levels (where data is available). We used the results from this first method to select specific forecasting methods to be further stress-tested over the most volatile demand periods using a similar approach by Yang et al. [3].

**Fig. 6:**
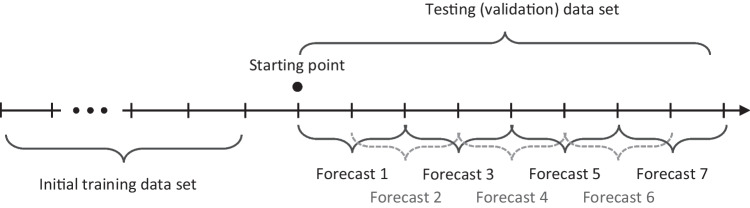
Expanding window cross-validation procedure

The first evaluation method uses all data up to a given day to train the model and then uses a forecast expanding window time horizon *t* to conduct in-sample cross-validation. We implemented this procedure using Rob Hyndman’s time series cross-validation function, tsCV(), in the R programming language [66]. Figure 5 shows how tsCV() works for an expanding window of 3-days ahead. Once a starting point is chosen, the model is trained using all previous time-series data (i.e., the initial training data) and a forecast will be made for a pre-defined forecast window for 3-days ahead (shown in Fig. 5 as “forecast 1”) with the first forecast at the starting point. The forecast window then moves one period forward and a second forecast window of the same time horizon is made (“forecast 2”). This process is repeated until the end of the data series. The size of the window can vary. We used an expanding window procedure with time horizons of *t* = 1, 2, 3 … 14 days. Forecast errors were calculated (i.e., the difference between the actual observation and the forecasted values) for each forecast window so comprehensive forecast error metrics (i.e., MAE and RMSE values) along with the benchmark error metrics could then be averaged over all the forecast windows (Fig. 6).

We further employed three additional metrics in this study to measure the accuracy of models within the expanding window cross-validation procedure against common benchmarks: (1) a naïve forecast method, (2) a 5-day centered moving average model (that was also used by Baas et al. [5]), and (3) a comparison of the prediction intervals (80%, 90%, 95%, and 99%) of various forecast methods relative to the current set capacity levels of the two COVID-19 wards (Red and Yellow). For our 3 benchmarks, we use the “mean absolute scaled error” (MASE) which is specifically designed to compare competing forecasting methods to assess accuracy. The MASE is calculated by dividing the MAE of a given model divided by the MAE of the benchmark model. For example, the MASE benchmark for a naïve forecast compares a given forecast method's MAE to a naïve forecaster's MAE. If the value of MASE is less than one, the forecasting method is more accurate on average than a naïve forecast method that simply uses a given day’s observation as the forecasted value in the next period.

Conversely, if MASE is greater than one, the naïve forecast model is more accurate than the given forecast method. MASE is considered a more appropriate metric to evaluate forecast accuracy for in-sample analysis when time-series observations are close to zero [59, 67]. Likewise, MASE avoids the issue associated with producing error metrics that are infinite or undefined due to having intermittent data as we have here [67]. There are times at St. Paul’s Hospital when the number of patients hits zero patients in the Red and Yellow COVID-19 wards and this will produce infinite or undefined values for some forms of forecast error metrics (i.e., the mean absolute percentage error). The second benchmark metric calculates MASE comparing the MAE of a given model to the MAE of a 5-day centered moving average model that averages the two previous day’s demand, the current day’s demand, and two future days’ demand. This method was also applied as a benchmark in similar research for ward-level forecast accuracy (in Baas et al. [5]). The final MASE benchmark is used to compare the accuracy of point forecasts with specified prediction levels (80–99%) against the error metrics defined by the planned capacity levels set by hospital staff for the COVID-19 wards. Hospital staff would tend to set capacity levels of COVID-19 wards high enough to provide a sufficient number of beds for any patient assigned to the Red or Yellow wards. Given that any forecast is considered to be the expected value (or mean) of the anticipated demand, we compared the planned capacity levels set by hospital staff to the upper forecast prediction intervals of 80%, 90%, 95%, and 99%. In doing so, we created our own variation of MASE, using MAE of the planned capacity levels (set by hospital staff) in the denominator, that indicates if a forecasting method with its specified prediction interval (80%-99%) is better or worse than the hospital staff's current capacity levels.

## Results

We focused on data from the second and third waves at both hospitals (Sept. 14, 2020, to June 29, 2021) as the cross-validation data set to compare the accuracy of competing forecast methods. This time period was selected as it was a period of considerable stress and volatility of the COVID-19 wards at both hospitals and represents a period during the pandemic with a high degree of diagnostic uncertainty. Forecasting error metrics were the largest for the combined total number of COVID-19 patients in the Red and Yellow wards; therefore, we present these results in this paper. Forecasting error metrics for the Red ward and the Yellow ward were consistently lower at both hospitals.^7^

Tables 5 and 6 demonstrate the results of the cross-validation of statistical and ML forecasting methods at SPH and VGH, respectively. Columns 3 and 4 show the absolute error metrics of RMSE and MAE, respectively (bolded values denote lowest errors), applied to expanding window forecasts of 1, 3, 5, 7, and 14 days ahead. Compared to other ward-level forecasting methods published in the literature [3, 5], the error metrics produced in this study are generally similar or lower, suggesting good accuracy of statistical and ML forecasting methods to ward-level forecasting. Generally speaking, ARIMA, ARIMAX, and NNARX demonstrate the lowest error metrics for longer time horizons of 5, 7, or 14 days ahead as shown by the bolded errors. For SPH, Table 5 shows that both statistical and ML point forecasts with defined prediction intervals can improve decision-making accuracy for capacity planning. All forecasting methods using point forecasts with increasing prediction intervals (80–99%) applied to the total number of COVID-19 patients (combined Red and Yellow wards) demonstrate a reduction in forecast error when compared to the error of the planned capacity levels that were set by hospital staff. As shown in the far-right column of Table 5, MASE is calculated by dividing the MAE of the various models (point forecasts with upper prediction levels) by the MAE of the planned capacities set in both the Red and Yellow wards (MAE = 9.77) using a 1-day ahead time horizon. For example, the MASE value for the most accurate forecasting method (ARIMAX) while using an upper prediction level of 95% is 0.52. This is calculated by dividing the MAE metric of the upper 95% prediction level for ARIMAX (5.04) by the MAE of the planned capacities set in the Red ward (9.77).^8^ MASE values less than one indicate an improvement in forecast accuracy relative to the benchmark whereas values greater than one indicate the benchmark method is more accurate. Notably, these forecasting methods improve forecast accuracy while simultaneously covering a high proportion of demand requirements for the COVID-19 wards. This is shown in the coverage rate (“CR”) column indicating how often the realized bed occupancy using an expanded window was covered by the 95% prediction interval. These results are consistent with the results of the Red and Yellow ward when analyzed independently.

**Table 5:** Cross-validation of statistical and machine learning forecasting methods at SPH compared to planned capacities (with prediction intervals) and benchmark forecasters for combined Red and Yellow wards. Bolded numbers depict the lowest forecast errors

Model	Expanding Window Forecasts	CR	Absolute Performance		Relative Performance using MASE		
			RMSE	MAE	vs Naïve	vs MA	vs Planned Capacity Levels^1^
SES	1d	0.97	**3.05**	1.56	0.87	0.98	(0.16; 0.33; 0.43, 0.52; 0.73)
	3d	0.94	3.97	2.00	0.90	0.97	
	5d	0.90	4.49	2.22	0.92	0.90	
	7d	0.86	4.98	2.36	0.87	0.85	
	14d	0.84	5.50	2.57	0.90	0.86	
Holts	1d	0.96	3.05	**1.55**	0.86	0.97	(**0.16**; 0.34; 0.44, 0.54; 0.75)
	3d	0.94	3.92	1.98	0.89	0.96	
	5d	0.89	4.58	2.24	0.92	0.91	
	7d	0.87	5.19	2.49	0.91	0.90	
	14d	0.85	6.13	2.93	1.03	0.98	
Holts, Damped	1d	0.96	3.05	1.57	0.88	0.99	(0.16; 0.33; 0.43, 0.52; 0.73)
	3d	0.94	3.87	1.97	0.88	0.95	
	5d	0.89	4.49	2.23	0.92	0.90	
	7d	0.87	4.98	2.36	0.87	0.85	
	14d	0.85	5.50	2.57	0.90	0.86	
ARIMA	1d	0.99	3.06	1.56	0.87	0.98	(0.16; 0.34; 0.43, 0.53; 0.73)
	3d	0.94	**3.86**	**1.97**	0.88	0.95	
	5d	0.94	4.45	2.24	0.92	0.91	
	7d	0.88	5.01	2.40	0.88	0.86	
	14d	0.90	5.45	2.57	0.90	0.86	
ARIMAX	1d	0.99	3.09	1.58	0.88	0.98	(0.16; **0.33**; **0.42**, **0.52**; **0.72**)
	3d	0.94	3.92	1.98	0.89	0.96	
	5d	0.95	**4.42**	**2.16**	0.89	0.88	
	7d	0.85	**4.83**	**2.30**	0.85	0.83	
	14d	0.86	**5.01**	2.31	0.81	0.77	
NAR	1d	0.94	3.46	1.74	0.97	1.09	(0.18**;** 0.35; 0.45, 0.55; 0.76)
	3d	0.90	4.46	2.24	1.00	1.08	
	5d	0.86	5.06	2.47	1.02	1.00	
	7d	0.85	5.80	2.81	1.03	1.01	
	14d	0.86	5.71	2.62	0.92	0.87	
NARX	1d	0.98	3.54	1.60	0.89	0.99	(0.16; 0.38; 0.49, 0.60; 0.84)
	3d	0.94	4.23	2.03	0.91	0.98	
	5d	0.91	4.76	2.21	0.91	0.90	
	7d	0.88	5.38	2.49	0.92	0.90	
	14d	0.91	5.09	**2.27**	0.80	0.76	

^1^The 5 MASE_M_ values for the Planned Capacity Levels correspond to the following: point forecasts (expected mean), upper 80% prediction interval, upper 90% prediction interval, upper 95% prediction interval, and the upper 99% prediction interval, respectively

Bolded numbers depict the lowest forecast errors

**Table 6:** Cross-validation of statistical and machine learning forecasting methods compared at VGH and benchmark forecasters for combined Red and Yellow wards. Bolded numbers depict the lowest forecast errors

Model	Expanding Window Forecasts	CR	Absolute Performance		Relative Performance	
			RMSE	MAE	vs Naïve	vs MA
SES	1d	0.98	3.47	1.18	0.95	0.88
	3d	0.93	5.04	1.60	0.96	0.85
	5d	0.90	6.32	1.89	0.96	0.90
	7d	0.83	7.36	2.08	0.95	0.94
	14d	0.79	10.29	2.74	0.95	0.95
Holts	1d	0.99	3.45	1.17	0.93	0.86
	3d	0.93	5.04	1.60	0.96	0.85
	5d	0.89	6.32	1.91	0.96	0.91
	7d	0.83	7.37	2.10	0.96	0.95
	14d	0.79	10.37	**2.72**	0.94	0.94
Holts, Damped	1d	0.99	3.50	1.20	0.96	0.89
	3d	0.93	5.08	1.61	0.97	0.86
	5d	0.89	6.34	1.90	0.96	0.91
	7d	0.83	7.37	2.08	0.96	0.95
	14d	0.79	10.28	2.75	0.96	0.95
ARIMA	1d	0.99	**3.35**	**1.12**	0.90	0.83
	3d	0.96	5.08	**1.59**	0.95	0.84
	5d	0.90	6.37	**1.88**	0.95	0.90
	7d	0.85	7.41	**2.07**	0.95	0.94
	14d	0.80	10.37	2.80	0.97	0.97
ARIMAX	1d	0.99	3.37	1.20	0.96	0.89
	3d	0.93	**4.92**	1.59	0.96	0.85
	5d	0.87	**6.17**	1.92	0.97	0.91
	7d	0.85	**7.11**	2.16	0.98	0.98
	14d	0.83	9.56	2.86	0.99	0.99
NAR	1d	0.98	3.71	1.27	1.02	0.94
	3d	0.90	6.26	1.84	1.10	0.98
	5d	0.85	7.89	2.15	1.09	1.02
	7d	0.81	8.68	2.23	1.03	1.01
	14d	0.72	11.34	2.92	1.01	1.01
NARX	1d	0.98	4.53	1.26	1.01	0.97
	3d	0.95	6.37	1.78	1.07	1.06
	5d	0.85	7.17	2.08	1.05	1.07
	7d	0.79	7.82	2.26	1.04	1.00
	14d	0.64	**9.47**	3.05	1.06	1.05

Bolded numbers depict the lowest forecast errors

The second benchmark metric shows that most forecasting methods using point forecasts are more accurate than a moving average forecaster for all expanding window time horizons as shown in Tables 5 and 6. The final benchmark method of using a naïve forecasting method within the expanding window cross-validation technique is the most rigorous of accuracy comparisons. This is simply because the naïve method will still utilize a 1-day step time horizon forecast within its expanding window, unlike the other forecasting methods that forecast the entire expanding windows before calculating the error metrics. Five out of the seven methods in both Tables 5 and 6 still demonstrate MASE values of less than one across all expanding window time horizons.

We also recognize the importance of comparing the RMSE values across the different forecast methods, as shown in Tables 5 and 6. The RMSE value provides greater sensitivity to larger forecast errors and for this reason, it may be a better indicator of the accuracy of the competing forecast methods. In our study, larger forecast errors would indicate a greater difference between the forecasted demand and the actual demand in the COVID-19 wards and, therefore should be heavier penalized for such errors. Having periods of large shortages or significant oversupply of beds and staffing within the COVID-19 wards would be extremely detrimental to both patient care and hospital employees. At both hospitals, ARIMAX and NNARX demonstrated the lowest RMSE error metrics for longer time horizons of 5, 7, and 14 days demonstrating the importance of external covariates to capture longer-term dynamics of ward-level demand.

### Selection of forecasting methods

Regarding which forecasting method works best, we need to discuss the application of the TCB protocol used for ward-level planning and the frequency of decision-making that occurs for expanding or contracting individual wards. This analysis can only be performed for SPH because data regarding the capacity level decisions of the wards at VGH were unavailable. During the cross-validation period (Sept 2020 to June 2021), SPH experienced 42 weeks of decision-making using the TCB protocol to manage the size of the COVID-19 wards (Red and Yellow). During that time, the frequency of decision-making to expand or contract the size of COVID-19 wards ranged from 0 times per week (at low periods during the pandemic) to 4 times per week (typically at times when numbers were increasing). On average, decisions relating to ward size occurred 1.38 times^9^ per week (or every 5.07 days). This indicates that the expanding forecasting window of 5 days is likely a good indicator of the forecasting method that would have closely modeled the decision-making frequency at SPH using the TCB protocol to manage the size of the COVID-19 wards. Using the RMSE error metric as a preferred error metric, given its greater sensitivity to large errors that occur, Table 5 indicates that ARIMAX, followed by ARIMA, provides the best overall accuracy for a 5-day expanding window for the total number of COVID-19 patients in the Red and Yellow wards. Notably, both methods provide MASE values of less than 1 when using point forecasts with increasing prediction intervals (80–99%) while simultaneously providing high coverage rates within the wards. Noticeably ARIMAX provides the best overall accuracy over longer time horizons of 7 and 14 days. As shown in Table 6, the cross-validation results are quite similar for VGH, which experienced greater demand volatility during the second and third waves relative to SPH.

In our efforts to develop an online forecasting tool for ward-level forecasting, we selected ARIMA and ARIMAX as the default methods for several reasons. The first reason is that both methods provide excellent forecast accuracy against competing methods, consistently outperforming benchmarks across all time horizons. Secondly, upper prediction intervals outperformed the accuracy of the planned capacity levels that were set by hospital staff at SPH, demonstrating the potential for improvement in decision-making for resource allocation of staffing and beds. Moreover, both methods are relatively simple to implement compared to a more complicated method such as NNAR, which does not readily produce prediction intervals. As discussed, we strive to implement a simple forecasting tool that can be used by healthcare professionals, requiring minimal input variables and no prior knowledge of forecasting to use it.

### Further accuracy metrics of selected models

**Fig. 7:**
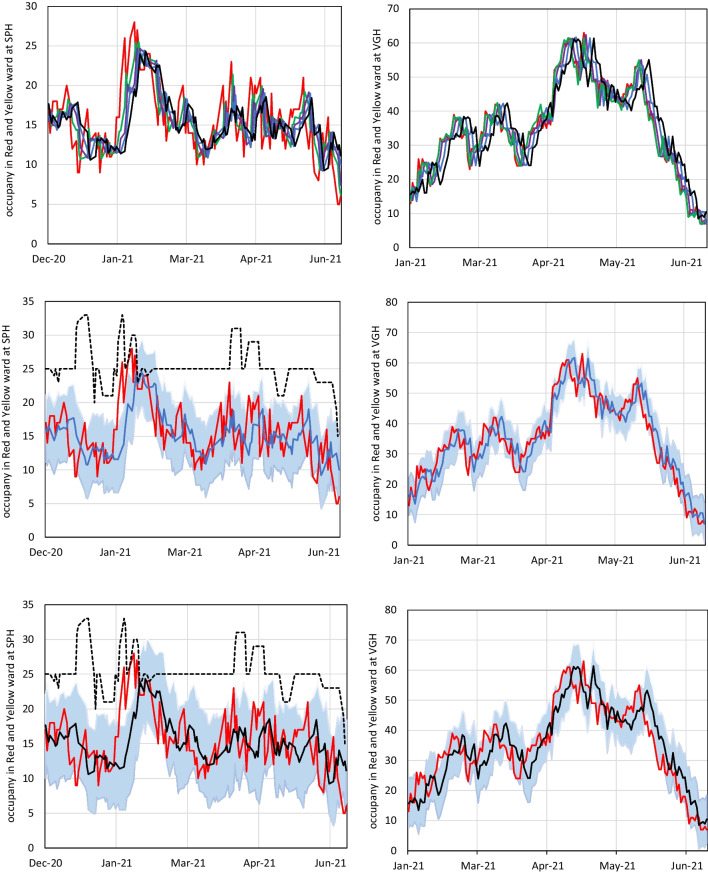
Cross-validation of the ARIMAX forecasting method at SPH and VGH during the greatest demand volatility of the second and third waves for each hospital. Top row: expanding window forecasts 1, 3, 5, and 7 days ahead in the combined COVID-19 red and yellow wards at SPH (left) and the combined COVID-19 red and yellow wards at VGH (right). Middle row: expanding window forecasts for 5 days ahead in the combined COVID-19 red and yellow wards at SPH with total planned capacities for the COVID-19 wards set by hospital staff (left) and the combined COVID-19 red and yellow wards at VGH (right), along with the 95% prediction interval. Bottom row: same as middle row except using expanding window forecasts for 7 days ahead

To further illustrate the accuracy of the selected forecasting methods, Fig. 7 shows the expanding window forecasts using an ARIMAX forecast method applied to both Red and Yellow wards to anticipate future bed occupancies during the cross-validation period. The top figure demonstrates the expanding window forecasts for 1, 3, 5, and 7 days ahead in the combined Red and Yellow wards at the SPH (left) and VGH (right) wards. As shown in the top row of this figure, the accuracy of the expanding window forecasts improves closer to the time of prediction. For example, the 1-day ahead forecasts (green) were more accurate to the actual demand (shown by the red line) than the 3-day ahead forecasts (purple), 5-day ahead forecasts (blue), and the 7-day ahead forecasts (black). Likewise, the middle row illustrates the accuracy of the 5-day ahead forecasts (blue line) along with a 95% prediction interval. Lastly, the bottom row demonstrates the 7-day expanding window forecasts’ accuracy and a 95% prediction interval. SPH (right) also includes the dashed black line representing the planned capacity levels set by hospital staff in the COVID-19 wards.

Figure 7 provides a few important revelations of the accuracy of these methods. First, the visualizations for SPH (left) demonstrate how using the upper 95% prediction interval of a 5-day ahead forecast would have produced an accurate benchmark to set capacity levels in COVID-19 wards (except for one period of extreme demand that took place in January 2021). Notably, using such a method to set capacity levels would have achieved consistently high coverage rates and would have likely provided a greater utilization of hospital resources within COVID-19 wards (also positively impacting external resources of COVID-19 wards). Second, we find that ARIMAX tends to have greater accuracy at the larger hospital (VGH) during periods of dramatic increases or decreases in patient demand. As shown, SPH (left) and VGH (right) demonstrate sustained periods of sharp increases in total ward-level demand in January 2021 and April 2021, respectively. At SPH, both ARIMA and ARIMAX tend to have a lagged response during periods of sharp increases or decreases of more than 10 patients over 5 days (i.e., inflection points). Comparatively, VGH’s point forecast and prediction intervals are more reactive to inflection points in ward-level demand during such periods. This is due to the stronger covariates found for VGH relative to SPH as discussed in Section 4.1. Lastly, it’s interesting to note that during periods of sharp increases in demand at VGH, the upper 95% prediction interval would have provided a valuable benchmark for ward-level planning. Likewise, during periods of sustained decreases at VGH, the point forecasts would have provided a valuable benchmark for ward-level planning.

To further demonstrate the performance of the selected forecasting methods (ARIMA and ARIMAX) that are used within our prototype forecasting tool, we use a similar approach used by Yang et al. [3] by forecasting the total number of COVID-19 patients with a time horizon of 7 days (on each Monday we make ward-level predictions for the next Monday), comparing with the actual value. This allows a direct comparison of other forecasting methods developed in research for the same purposes. We selected two 10-week periods at both hospitals during the second and third waves, demonstrating the greatest volatility of ward-level patient demand for this analysis.^10^ These peaked time periods occur at different times demonstrating that each hospital experienced a variation in patient demand despite being located within the same municipality. This demonstrates the importance of using localized forecasting at the ward level within each hospital due to each having its specific demand characteristics.

**Fig. 8:**
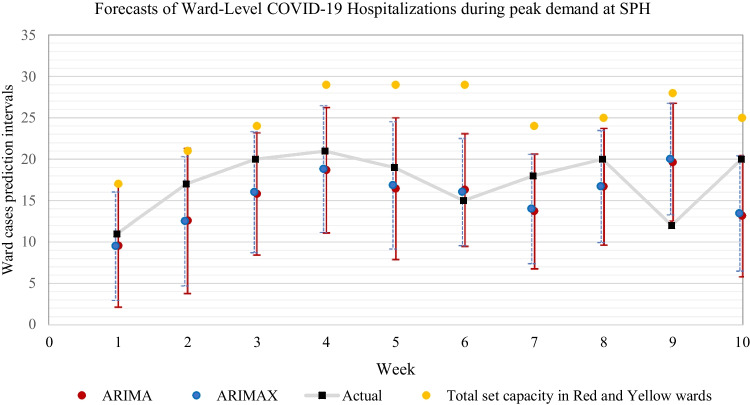
SPH ward-level forecasts, time horizon = 7 days, 95% prediction intervals, with black squares representing actual values

Here we present the results of one of the 10-week periods for each hospital that demonstrated the poorest forecast accuracy. It should also be pointed out that the volatility experienced by SPH seems similar to the medical wards used by Yang et al. [3], and VGH’s ward-level hospitalizations show a higher degree of volatility, with peak demands being twice that of SPH. As shown in Fig. 8, the 95% upper prediction interval of both ARIMA and ARIMAX consistently provides better accuracy than set capacities at SPH for the total number of beds in the Red and Yellow wards. Both models provided a 90% coverage rate across 10 weeks of weekly forecasting during the greatest volatility of total ward-level demand. As expected, SPH shows little to no difference between ARIMA and ARIMAX methods providing further evidence that covariates at smaller hospitals may not be as effective inputs for forecasting ward-level demand compared to larger hospitals that are likely to experience a greater association with community-level epidemiological factors.

**Fig. 9:**
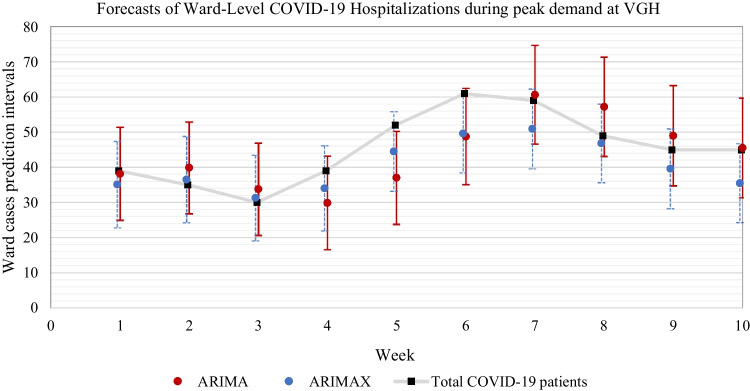
VGH ward-level forecasts, time horizon = 7 days, 95% prediction intervals, with black squares representing actual values

**Fig. 10:**
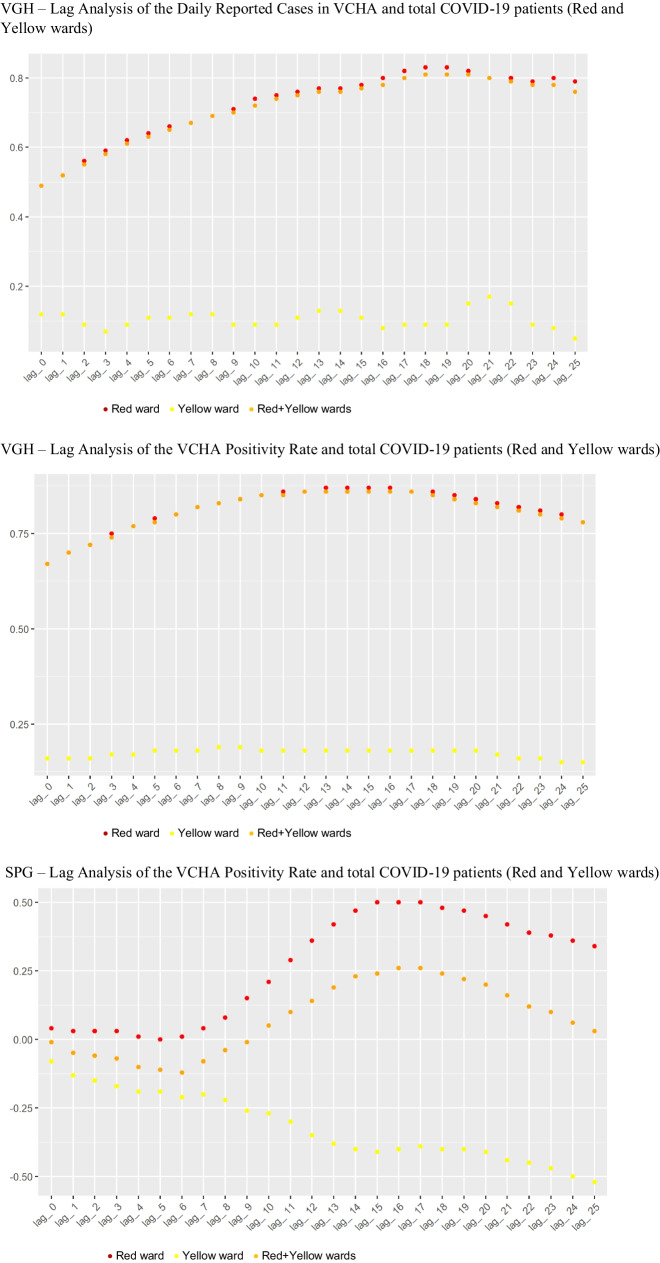
VGH – Lag Analysis of the Daily Reported Cases in VCHA and total COVID-19 patients (Red and Yellow wards). VGH – Lag Analysis of the VCHA Positivity Rate and total COVID-19 patients (Red and Yellow wards). SPG – Lag Analysis of the VCHA Positivity Rate and total COVID-19 patients (Red and Yellow wards)

The results are quite different for the larger hospital (VGH) across its most volatile demand period. As shown in Fig. 9, the 95% prediction interval of ARIMAX is more reactive during weeks of increasing or decreasing trends, where ARIMA tends to have a slightly lagged effect and has periods of overshooting on upward trends and delays on downward trends. Ideally, forecasting methods should strive to provide 100% coverage of patients needing care while simultaneously minimizing the number of unused beds. The result show that the upper 95% prediction interval of ARIMAX is better positioned to achieve these objectives simultaneously compared to the upper 95% prediction interval of ARIMA. Despite this, both methods provide excellent coverage rates of the actual number of patients, similar to that of Yang et al. [3].

As shown in Table 7, the fraction of the weeks for which the 95% prediction intervals of ARIMA and ARIMAX covered the observed bed count at VGH during the highest period of peak demand is 90% and 100%, respectively. Likewise, the error metrics are generally low with ARIMAX showing greater accuracy for both point forecasts and the upper 95% prediction intervals compared to ARIMA.

**Table 7:** Weekly forecast error metrics and coverage rates of point forecasts and the 95% prediction intervals over 10 weeks of the greatest volatility in ward-level demand at SPH and VGH

		Point Forecast Accuracy		Upper 95% PI Accuracy	
SPH	Coverage Rate	RMSE	MAE	RMSE	MAE
ARIMA	90%	4.34	3.84	6.57	5.44
ARIMAX	90%	4.27	3.72	6.34	5.15
VGH					
ARIMA	90%	7.64	6.02	12.56	14.41
ARIMAX	100%	6.48	5.57	6.68	8.00

This analysis demonstrates that weekly ward-level forecasting can be accurately anticipated with weekly changes of approximately 10–15 patients (increase or decrease) captured by both smaller (SPH) and larger hospitals (VGH). Overall, the results show that using an upper 95% prediction interval with weekly forecasts can improve decision-making with setting capacity levels. It also shows the fact that larger hospitals are likely to experience greater accuracy with using statistical forecasting methods by including external covariates (epidemiological community factors) relative to smaller hospitals.

## Discussion and conclusion

Past research has primarily focused on forecasting methods applied to larger regional or country-level growth patterns to predict aggregate demand for medical resources during the COVID-19 pandemic. As discussed in this paper, hospital wards experience unique demand patterns for COVID-19 patients that vary across geographical regions and even between hospitals within the same health authority. Thus, we argue that the operational planning of hospital wards requires a forecasting approach vastly different from the population growth models well documented in the literature for pandemic forecasting [49, 51]. Ward-level forecasting methods help resource planning decisions during the earliest stages of the COVID-19 pandemic on two levels. One is related to the total COVID-19 ward space dedicated in a given hospital, and the other is related to the expansion and contraction of interdependent COVID-19 wards within the total dedicated ward space. In this paper, we add to the few forecasting methods in the literature that have been effectively applied to ward-level forecasting that support pandemic relief, particularly to aid with planning of hospital staffing that has experienced dramatic burn-out during the COVID-19 pandemic.

This paper is the first to explore the use of statistical and ML forecasting methods and their accuracy in forecasting ward-level demand to aid in planning of hospital resources (beds, staffing, etc.) during the COVID-19 pandemic. Our results confirm that traditional statistical and ML forecasting methods can provide valuable ward-level forecasting to aid in decision-making for pandemic resource planning. Using point forecasts with upper 95% prediction intervals, such forecasting methods would have provided better accuracy in anticipating required beds in both COVID-19 wards (confirmed positive and PUI wards) than ward-level capacity decisions made by hospital staff during the second and third waves of the pandemic. From the perspective of hospital staff in COVID-19 wards, this translates into more accurate decision-making in setting ward capacity levels during pandemics. The cost of over or under-allocating beds in COVID wards is high. Over-allocation leads to idle physicians and nurses (lost wages), reduced capacity in non-COVID-19 areas, and potentially its impacts on additional off-site wards in extreme cases of a pandemic. Under-allocation leads to physician, nurse, and allied health staff burn-out, impaired ability to maintain infection control precautions (increased risk of nosocomial transmission), patient morbidity, and mortality in severe cases.

The use of specific statistical and ML forecasting methods for ward-level forecasting is appropriate under specific conditions related to forecasting accuracy, benefits, and limitations. Regarding forecasting accuracy, we find that ARIMA, ARIMAX, and NARX provided the best overall accuracy among the various forecast methods analyzed for two hospitals in this study for ward-level forecasting for 5, 7, and 14 days ahead time horizons. NARX, although it provided good accuracy, was removed from consideration due to its challenges of producing prediction intervals and its sensitivity to missing values which can be quite common. ARIMA and ARIMAX are easier to implement within our online forecasting tool and are well-documented in literature as effective time-series forecasting methods across many healthcare applications [68]. For example, Sun et al. [69] found that ARIMA provided good forecasting accuracy for daily patient attendance at ED in Singapore even though daily variations were quite significant, ranging from 10 to 72 patients per day. Likewise, these authors found similar improvements in the forecasting performance of ARIMA when combined with other variables like air quality and public holidays. For pandemic forecasting at the ward-level, ARIMA has the added benefit that it does not require specialized modeling requirements relative to other ward-level forecasting methods that have recently been published in the literature (e.g., regional forecast requirements [3], complicated queueing models, or probabilistic modeling [3, 51]). Diagnostic and validation tools for ARIMA and ARIMAX can also be implemented in our online forecasting tool for those users that are more well-versed in forecasting methods. This study shows that ARIMA and ARIMAX are accurate for ward-level forecasting for moderate changes (increases or decreases) of approximately 10–15 patients every 3 or 4 days. This degree of volatility is likely to be representative of the ward-level volatility across many hospitals that utilized a modified TCB protocol during the pandemic.^11^ Such forecasting methods would not be suitable for explosive changes in demand, such as monotonically increases or decreases over time, that would likely be better modeled using population growth curves as shown in the literature for regional changes. Likewise, statistical and ML methods would fail to anticipate ward-level demands in large increases that would happen overnight without having prior data to identify such trends. Such forecasting methods are inherently limited by past time-series patterns and therefore data quality of both COVID-19 patient numbers and any external covariates are requirements for such methods to provide valuable inputs to ward-level decision-making.

In this study, we found that the ward-level demand of COVID-19 patients in the largest hospital in British Columbia, Canada (VGH) was highly correlated with time-lagged epidemiological factors, such as positivity rates and daily reported cases within the local health authority (VCHA). We did not find such high correlations with epidemiological factors at a smaller hospital (SPH) within the same health authority. Such associations are valuable inputs for ARIMAX and NNARX forecasting methods in terms of overall forecast accuracy, particularly for longer time horizons of 7 and 14 days ahead. These results show that statistical and ML forecasting methods with strong covariates are likely to be more useful for ward-level forecasting at larger hospitals that are reflective of community-level pandemic effects.

In order to truly benefit from the findings of this study, we have built a working prototype forecasting tool using a package called Shiny as part of the R statistical software. The tool is still in development and requires improvements in terms of its usability for hospital staff for everyday use. A prototype is now publicly available online only for purposes of research assessment and dissemination: https://stpaulhospital.shinyapps.io/hospital_planning/

**Fig. 11:**
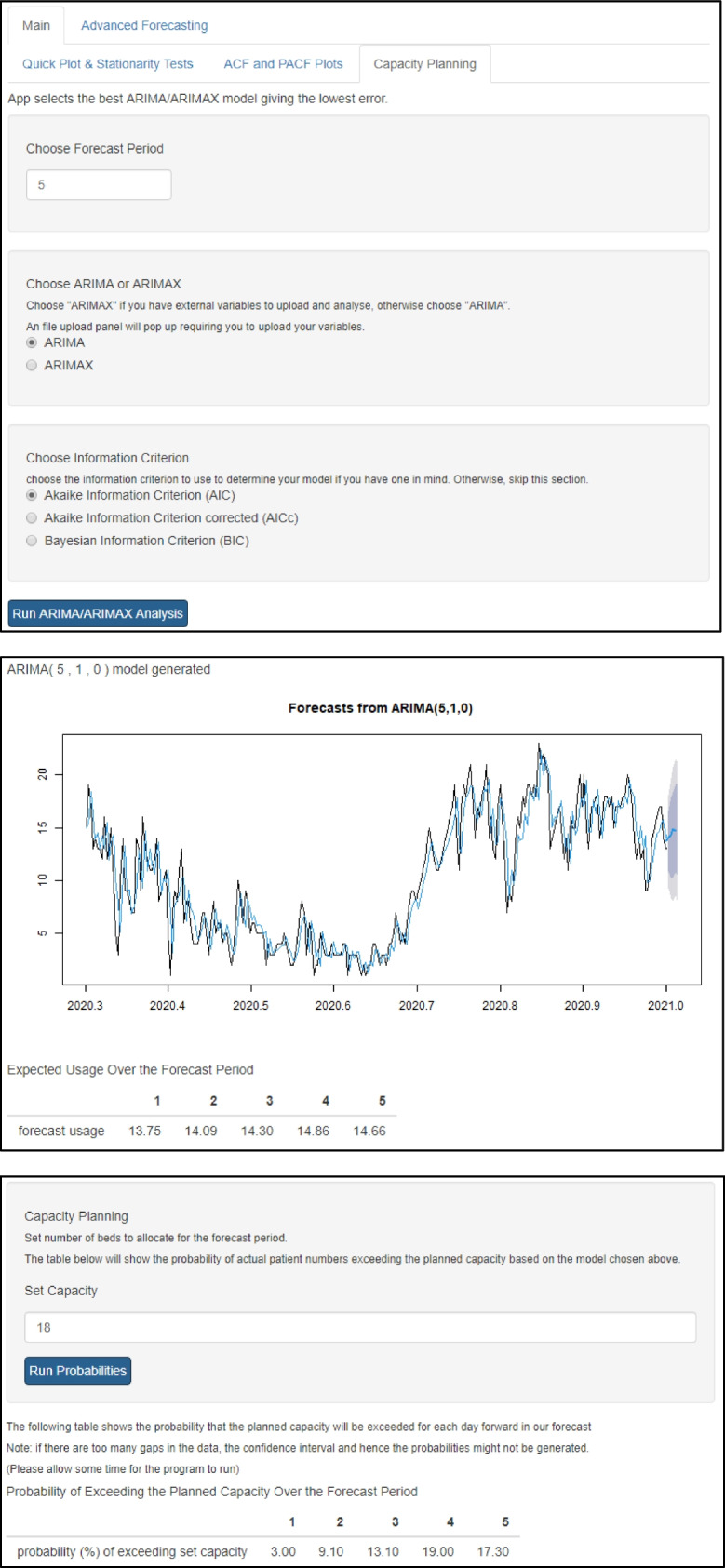
Capacity planning module integrated within a ward-level forecasting tool

As shown in Fig. 11 in Appendix 2, individuals can load data and forecast bed requirements to make informed decisions for individual wards within hospitals. It has been designed to provide real-time forecasting for the number of beds required in COVID-19 wards (Red), wards dedicated to PUI (Yellow), or the combined demand for both wards. The tool requires only the data to load and uses an ARIMA or ARIMAX default forecasting method to predict hospital ward demand. We also programmed the allowance for external covariates for improved forecasting to benefit from the improved accuracy found in longer time horizons with ARIMAX. The tool also has a capacity planning module that allows the user to determine the probability of exceeding a set capacity level within a forecast time horizon. The tool predicts both the forecasted values along with a 95% prediction interval using a bootstrapping method. The capacity planning tool uses the upper 95% prediction interval to calculate the probability that demand will exceed set capacity levels. The point of having the capacity planning module in the forecasting tool allows for risk analysis of coverage rates and unused bed capacity to be conducted. The trade-off between having too many beds and not enough beds for 100% patient coverage should ultimately be left in the hands of the hospital staff who make the decisions to expand or contract covid-19 wards, who best understand the immediate consequences of these two contradictory objectives. The forecasting tool’s capacity planning module provides the percentage likelihood of having a sufficient number of beds over the next 14 days which ultimately allows this risk to be analyzed. In periods of sustained increases of patient demand, having some drop in the coverage rate from 100% is likely to be warranted. For example, hospital staff at a larger hospital may decide that a 95% coverage rate is suitable given their ability to rapidly add more beds whereas at a small hospital any drop of 100% coverage may be deemed unacceptable. Likewise, most hospitals, during periods of decreasing demand, having too many unused beds in COVID-19 wards would likely be less concerning, relatively speaking.

Fig. 11 (in Appendix 2) provides an example of forecasting the required demand for the combined Red and Yellow wards with a set capacity for a total of 18 beds required. The capacity planning tool predicts the probability that demands within both wards will be exceeded over the next five days. As shown, the tool predicts an increasing likelihood that the capacity will be exceeded with only a 3% likelihood for the next day but a 19% likelihood on day 4. We believe this type of real-time feedback during decision-making on specific ward capacity is likely to be extremely valuable from a planning and cost perspective.

This work has several limitations and opportunities for future research. Our findings are limited to the medicine wards at two urban hospitals in Vancouver, Canada (one small to moderate-sized and the other a large hospital). Future research is to validate our forecasting tool at other hospitals across Canada and to improve the usability of the tool for hospital staff. We also intend to investigate the possibility of implementing alternative forecasting methods beyond the methods investigated in this study as more hospital data becomes readily available. It may be possible to identify more accurate forecasting methods or procedures that could improve the overall accuracy of ward-level predictions. Likewise, we would like to investigate further the relationship between time-lagged covariates and ward-level demand at other hospitals to identify if similar patterns found in this research can be replicated for both smaller and larger hospitals. We also plan to investigate the predictability of forecasting methods for greater time horizons and other specific periods within the pandemic, which relates to growing variant and vaccination rates. Further research may also investigate a number of external variables that may improve the predictability of statistical and ML forecasting methods, including the effect of hospital discharges, length of stay, and the number of admissions within the emergency department. Lastly, there is an opportunity to explore the repurposing of our forecasting tool beyond the pandemic to other scenarios such as the impact on ward-level demand during the flu-season from respiratory illnesses or the impact on ER bed demand during heightened seasonal periods.

## Appendix 1

 10

## Appendix 2

 11

## Footnotes

1 For example, the forecasting system produces accurate predictions of the total number of patients in COVID-19 wards (see Figs. 1 and 2 for time-series plots) with an average daily demand of approximately 30 beds with a standard deviation of 12 beds per day during the second and third waves of the pandemic.

2 COVID-19 positive, COVID-19 negative and COVID-19 patients under investigation (PUI).

3 At SPH, COVID-19 patient counts are calculated for the Red ward alone and does not include COVID-19 patients elsewhere in the hospital. At VGH, COVID-19 counts represent all patients in hospital admitted to Internal Medicine and therefore there are times shown where the number of patients exceed ward capacity.

4 Unfortunately, data on the set capacity levels of the Yellow ward at VGH were unavailable.

5 Correlation between positivity rate using a lag of 14 days and the Red ward demand increases to 0.73 across the entire first three pandemic waves.

6 We also implemented several seasonal statistical models including Holt-Winters (ETS), TBATS and Prophet that all proved inferior to the selected models. As indicated, our exploratory analysis demonstrated a negligible degree of seasonality across the time-series.

7 Error metrics for the Red ward were up to 20% lower, while error metrics for the Yellow ward were between 35 and 50% lower.

8 Another way we will present forecast accuracy in this paper will be to reverse the numerator and the denominator of the MASE calculation. For example, 9.77/5.04 = 1.93, therefore we can state that using an upper 95% prediction interval of an ARIMAX forecast method is 1.94 times more accurate on average than the planned capacity levels set by hospital staff during this validation period.

9 58 times over the 42 weeks.

10 For SPH, the greatest volatility (upward and downward trends) is from Sept 14/2020 to Nov. 23/2020 and from Dec. 28/2020 to Mar. 7/2021. For VGH, the greatest volatility in ward-level patient demand occurred from Nov. 1/2020 to Jan 10/21 and from Mar. 8/2021 to May 31/2021.

11 For example, the average weekly standard deviation of bed demand in the combined Red and Yellow wards at VGH over 42 weeks during the second and third waves was 3.3 beds per week (or, an average absolute range of approximately 8 patients weekly).
